# Optimization of Magnetic Abrasive Finishing Parameters for Co–Cr Alloy Vascular Stent Tubing Using PSO-SVM

**DOI:** 10.3390/mi17080916

**Published:** 2026-07-30

**Authors:** Kai Xing, Yugang Zhao, Qilong Fan, Li Guo, Zhi Qi, Kaihao Ma, Guangzheng Chen

**Affiliations:** School of Mechanical Engineering, Shandong University of Technology, No. 266 Xincun West Road, Zibo 255049, China; sdlgxingkai@126.com (K.X.); fql0317@126.com (Q.F.); guoli3225@126.com (L.G.); sdtuqizhi@126.com (Z.Q.); m1779014682@126.com (K.M.); c13044077615@126.com (G.C.)

**Keywords:** magnetic abrasive finishing (MAF), orthogonal experiment, support vector machine (SVM), particle swarm optimization (PSO), surface roughness

## Abstract

To achieve accurate prediction of surface roughness (Ra) in magnetic abrasive finishing (MAF) of the inner wall of Co–Cr alloy vascular stent tubing, and to obtain the optimal process parameter combination for improving the inner surface quality, iron-based diamond magnetic abrasive powders (MAPs) were prepared via plasma melting, centrifugal spraying and rapid solidification. MAF experiments were conducted on Co–Cr alloy vascular stent tubing with an inner diameter of 1.6 mm and an outer diameter of 1.8 mm, and the effects of tube rotational speed, magnetic pole feed rate, abrasive particle size and working gap on surface roughness were investigated. An orthogonal experiment was designed, and a surface roughness prediction model based on particle swarm optimization (PSO) and support vector machine (SVM) was established. Simulation results indicate that the proposed PSO-SVM surface roughness prediction model achieved a coefficient of determination (R^2^) of 0.96771, a root-mean-square error (RMSE) of 0.0012756 μm, and a mean absolute percentage error (MAPE) of 1.061%. The optimal parameter combination obtained by PSO-SVM optimization was a tube rotational speed of 832.6384 r·min^−1^, a magnetic pole feed rate of 129.6784 mm·min^−1^, a working gap of 0.5324 mm, and an abrasive particle size of 132.4185 µm. Under these conditions, the experimentally obtained surface roughness was 0.0949 μm, with a relative error of 0.58% compared to the model-predicted value. The results demonstrate that the established PSO-SVM surface roughness prediction model possesses favorable predictive capability, and its combination with MAF technology enables superior surface quality.

## 1. Introduction

Cardiovascular disease (CVD) remains the leading cause of morbidity and mortality worldwide, with China ranking first in global CVD-related deaths and bearing a particularly heavy disease burden [1,2,3]. Percutaneous coronary intervention (PCI), characterized by its minimal trauma, rapid postoperative recovery and reliable therapeutic efficacy, has emerged as the core interventional strategy for coronary artery stenosis and occlusion. As the critical implantable device in PCI procedures, the cardiovascular stent directly determines surgical success and long-term patient prognosis [4,5,6]. With the continued advancement of clinical interventional therapy toward complex lesions and precision medicine, increasingly stringent requirements have been imposed on the comprehensive performance of cardiovascular stents. These devices are required to be implanted in human coronary arteries for extended periods, necessitating an optimal combination of superior mechanical support, favorable biocompatibility and exceptional surface quality. Of particular importance is the inner wall of the tubing, which is in direct contact with blood; its surface roughness, microscopic defects and burrs can directly influence blood rheological behavior, promote platelet adhesion and aggregation, and consequently increase the risk of thrombosis and intimal hyperplasia. In severe cases, these surface imperfections may even lead to life-threatening complications, including in-stent restenosis and acute myocardial infarction. Moreover, given the complex anatomical structure and small caliber of coronary vessels, stents must exhibit favorable flexibility and deliverability to navigate tortuous vessels and reach the target lesion, thereby achieving effective support for stenotic arteries. These clinical requirements impose extremely high demands on the machining precision and surface integrity of stent substrate materials [7,8].

Cobalt–chromium (Co–Cr) alloys exhibit excellent corrosion resistance and favorable biocompatibility. In the complex ionic environment of human blood, they effectively resist corrosion and inhibit the release of metal ions, thereby avoiding inflammatory responses and immune rejection triggered by ion release, and substantially enhancing the long-term biosafety of stents. Furthermore, owing to their relatively high density, Co–Cr alloys enable clear radiopaque visualization during interventional procedures without the need for additional radiopaque coatings, facilitating precise stent deployment and positioning by clinicians while reducing procedural difficulty and associated risks. In addition, Co–Cr alloys possess higher tensile and yield strengths, which allow for ultra-thin strut design of stent scaffolds. By virtue of these comprehensive and superior material properties, Co–Cr alloys have progressively replaced conventional 316L medical-grade stainless steel, establishing themselves as the mainstream substrate material for contemporary clinical drug-eluting stents [9,10].

Despite the excellent material properties of Co–Cr alloys for cardiovascular stents, the stent tubing is characterized by a small inner diameter (typically < 2 mm), a high aspect ratio and an ultra-thin wall, rendering conventional machining processes inadequate for meeting the stringent requirements of high-precision and high-integrity finishing of the inner surface [11]. Conventional mechanical polishing employs rigid tools that cannot access the narrow inner wall, inevitably leading to processing blind zones; moreover, the process tends to generate scratches and stress layers that compromise the substrate integrity of the tubing, making it difficult to ensure consistent surface quality. Electrochemical polishing, although capable of improving surface roughness, suffers from issues such as over-etching, poor machining uniformity and heavy metal contamination, which do not comply with the green manufacturing requirements for medical devices, and it is also ineffective at removing deep-seated defects such as micro-cracks. Abrasive flow machining is limited by low processing efficiency and the difficulty of completely removing residual abrasives, which may cause serious complications such as vascular embolism, thereby compromising the safety of stent implantation. Laser processing, despite its capability for high-precision surface micro-texturing, may induce thermal effects that alter the surface metallurgy and corrosion resistance of Co–Cr alloys [12,13]. The inherent limitations of these conventional processes result in residual manufacturing defects, including burrs, tool marks and micro-cracks on the inner wall of Co–Cr alloy stent tubing, making it difficult to meet the surface roughness requirements for clinical hemocompatibility. According to ISO 10993 and FDA regulatory guidance for cardiovascular stents [14,15], the surface quality of implantable stents must satisfy the following requirements: surface roughness Ra ≤ 0.1 μm to achieve optimal hemocompatibility; the absence of surface defects such as cracks, burrs, and pits that may initiate thrombosis; and uniform surface characteristics to ensure controlled drug release and stable endothelialization. These stringent criteria necessitate advanced finishing technologies capable of achieving defect-free, ultra-smooth inner wall surfaces on small-diameter stent tubing. It is worth noting that although the outer surface of the stent tubing also has a certain influence on crimping behavior, balloon expandability, and tissue interaction after implantation, the present study focuses exclusively on the inner wall surface. This is because the inner wall is in direct contact with blood flow and is the most critical surface for hemocompatibility and thrombosis prevention. Meanwhile, due to the restricted accessibility, the finishing of the inner wall is far more challenging than that of the outer surface, whereas the outer surface can be more readily finished by conventional techniques such as mechanical polishing or electrochemical polishing, and is therefore not the main bottleneck in stent manufacturing.

Magnetic abrasive finishing (MAF), as a novel flexible precision finishing technology, demonstrates unique advantages in addressing the aforementioned challenges. This technology harnesses magnetic field forces to drive magnetic abrasive particles, forming a flexible magnetic brush that generates controlled micro-cutting and lapping actions on the workpiece surface, thereby enabling micro-scale material removal and surface smoothing. Compared with conventional methods, MAF offers three irreplaceable advantages: (1) excellent process flexibility—the magnetic abrasive particles can self-adaptively deform to conform to complex inner wall contours, making them particularly suitable for small-diameter, high-aspect-ratio tubular structures without creating processing blind zones; (2) controllable material removal—the normal force exerted by the abrasive particles on the wall surface can be precisely regulated by adjusting the magnetic field intensity and process parameters, avoiding over-processing or surface damage; (3) absence of tool wear and thermal effects—the magnetic brush is a flexible body with no rigid tool wear issues; the process generates minimal temperature rise and does not alter the surface properties of the material, making it especially suitable for heat-sensitive biomedical materials [16,17,18]. These characteristics endow MAF with significant application potential in the internal surface finishing of micro-medical devices.

To achieve mirror-like polishing of the inner wall of ultra-fine and slender Co–Cr alloy stent tubing, iron-based diamond magnetic abrasive powders (MAPs) with different particle sizes were first prepared via plasma melting, centrifugal spraying and rapid solidification. Subsequently, a dedicated MAF apparatus for the inner wall of cardiovascular stent tubing was designed and constructed. The effects of tube rotational speed, magnetic pole feed rate, abrasive particle size and working gap on surface roughness were then investigated. Based on the orthogonal experimental results, a PSO-SVM surface roughness prediction model was established. According to the model fitting results, the interactions among the process parameters were analyzed, and the process parameters were optimized using the established model and multiple regression equations. Finally, with surface roughness employed as the fitness function, the optimal process parameter combination for MAF of the inner wall of Co–Cr alloy vascular stent tubing was obtained through experimental verification, which significantly reduced the surface roughness and ultimately achieved mirror-like polishing of the inner wall of Co–Cr alloy stent tubing.

## 2. Preparation of Magnetic Abrasive Powders via Plasma Melting, Centrifugal Spraying and Rapid Solidification

### 2.1. Preparation Principle and Equipment for Magnetic Abrasive Powders

Magnetic abrasive powders (MAPs) are the core tool in MAF, and their performance plays a decisive role in determining the machining quality and polishing efficiency of the process [19]. Plasma melting, centrifugal spraying and rapid solidification represent a novel approach for preparing high-performance MAPs. The MAPs fabricated by this method are characterized by a robust bonding between the hard abrasive particles and the metallic matrix, with the hard abrasives predominantly distributed in the superficial layer of the iron-based matrix, while exhibiting high magnetic permeability and structural strength. These features make the prepared MAPs particularly suitable for addressing the polishing challenges associated with the inner wall of Co–Cr alloy vascular stent tubing.

**Figure 1:**
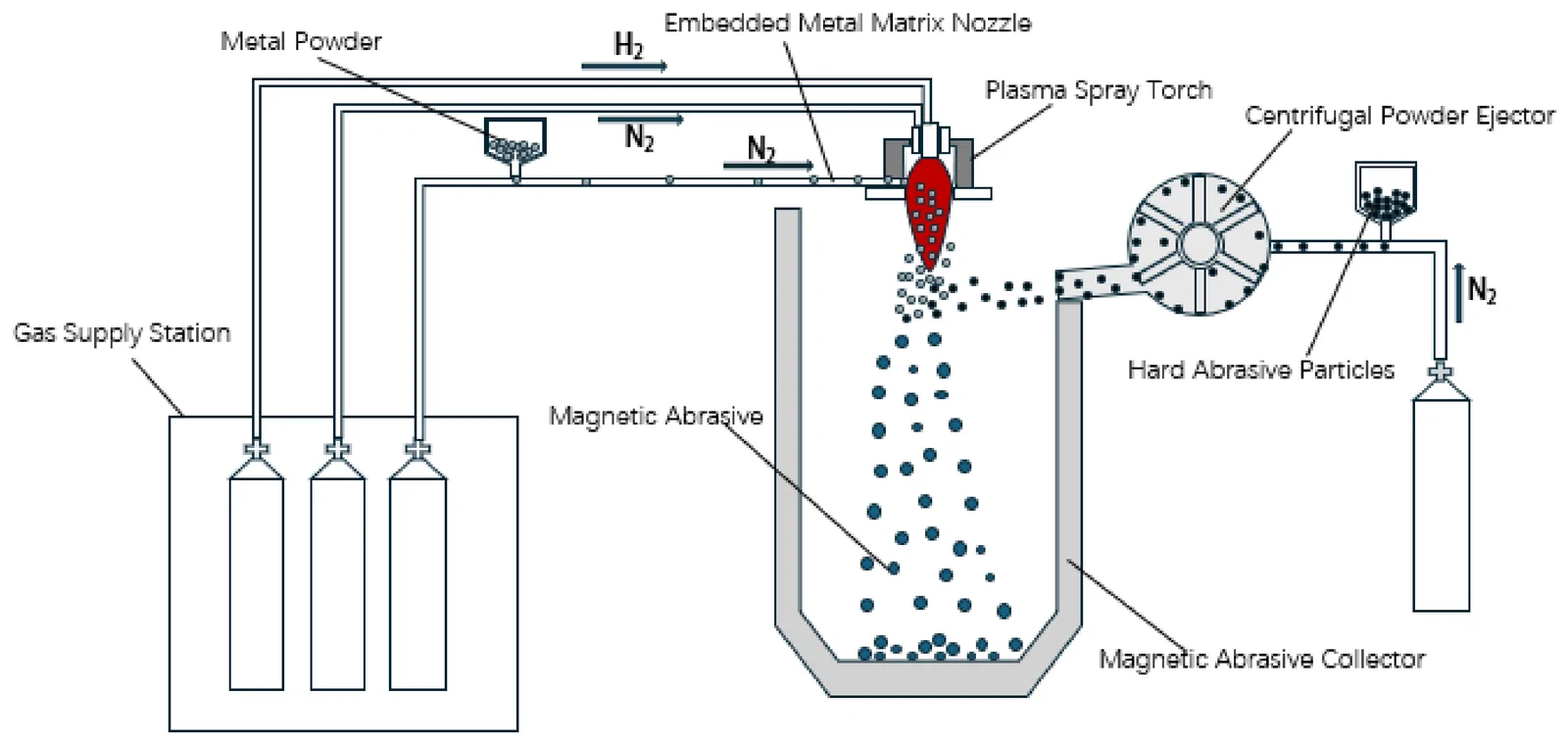
Schematic diagram of magnetic abrasives prepared by plasma melting and centrifugal powder ejection rapid solidification.

Figure 1 illustrates the preparation principle of magnetic abrasive powders via plasma melting, centrifugal spraying and rapid solidification. The equipment employed for this process primarily consists of a plasma generation system, a centrifugal powder sprayer, a spiral powder feeding system for the metallic matrix, a condensation chamber for magnetic abrasive powders, and a powder collection unit. High-frequency plasma is utilized to heat and melt metal powder particles of uniform particle size. During the descent of the molten metal micro-droplets, hard abrasive particles are injected into the plasma flame, enabling them to overcome the surface tension of the molten micro-droplets and penetrate into their interior. Before the hard abrasive particles can escape from the molten metal micro-droplets, rapid condensation occurs, yielding magnetic abrasive powders characterized by uniform particle size distribution, robust bonding between the hard abrasives and the metallic matrix, regular spherical morphology, and strong grinding and polishing performance.

### 2.2. Preparation of Spherical Iron-Based Diamond Magnetic Abrasive Powders

**Figure 2:**
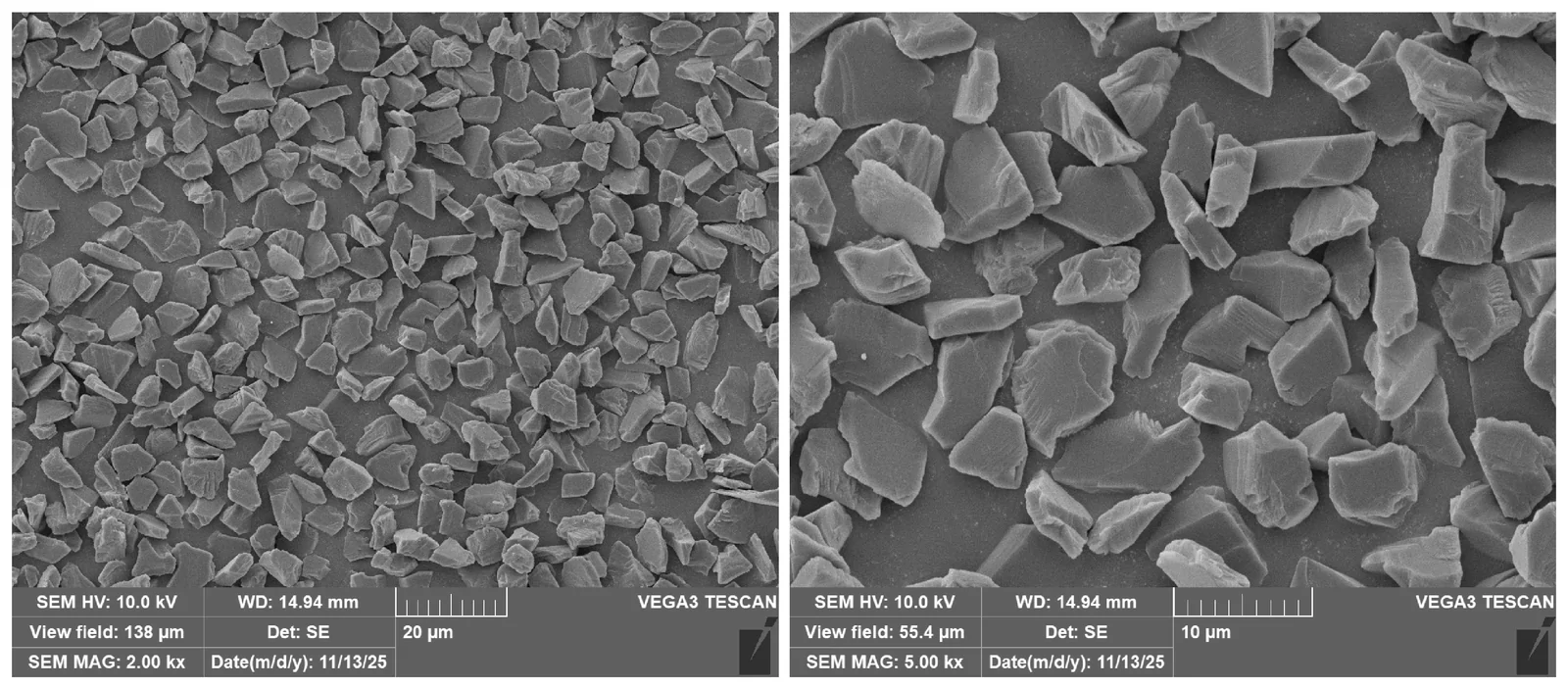
Scanning electron micrograph of diamond hard abrasives.

Diamond exhibits an exceptional microhardness of up to 10,000 kgf/mm^2^, along with outstanding thermal conductivity, high chemical stability, low thermal expansion coefficient and low friction coefficient [20,21,22]. Consequently, in comparison with conventional SiC and Al_2_O_3_ abrasives, diamond was selected as the hard abrasive phase for the preparation of magnetic abrasive powders, enabling superior polishing performance for difficult-to-machine materials. The SEM micrograph of the diamond hard abrasive particles is presented in Figure 2.

The procedure for preparing the iron-based diamond magnetic abrasive powders is as follows. First, the iron-based metallic matrix powders and diamond abrasive particles with specific size ranges were loaded into the metal powder feeder and the spiral powder feeder, respectively. Subsequently, the gas supply valves for the metal powder feed gas and the working gas were opened, and the power supply was activated. The gas flow rate, gas volume, working current and working voltage were then adjusted via the control interface to ensure stable operation of the abrasive preparation experiment. Upon completion of the experiment, the power supply and gas supply valves were immediately turned off, and the prepared magnetic abrasive powders were collected from the powder collection unit. It is worth noting that the plasma melting and rapid solidification process merely heats the particles to a molten state and then rapidly solidifies them, without altering the intrinsic size of the constituent materials. The collected magnetic abrasive powders were then classified using standard sieves, sealed and stored in a dry environment. Throughout the entire preparation process, the parameters listed in Table 1 and Table 2 were adopted for the fabrication of the iron-based diamond magnetic abrasives.

**Figure 3:**
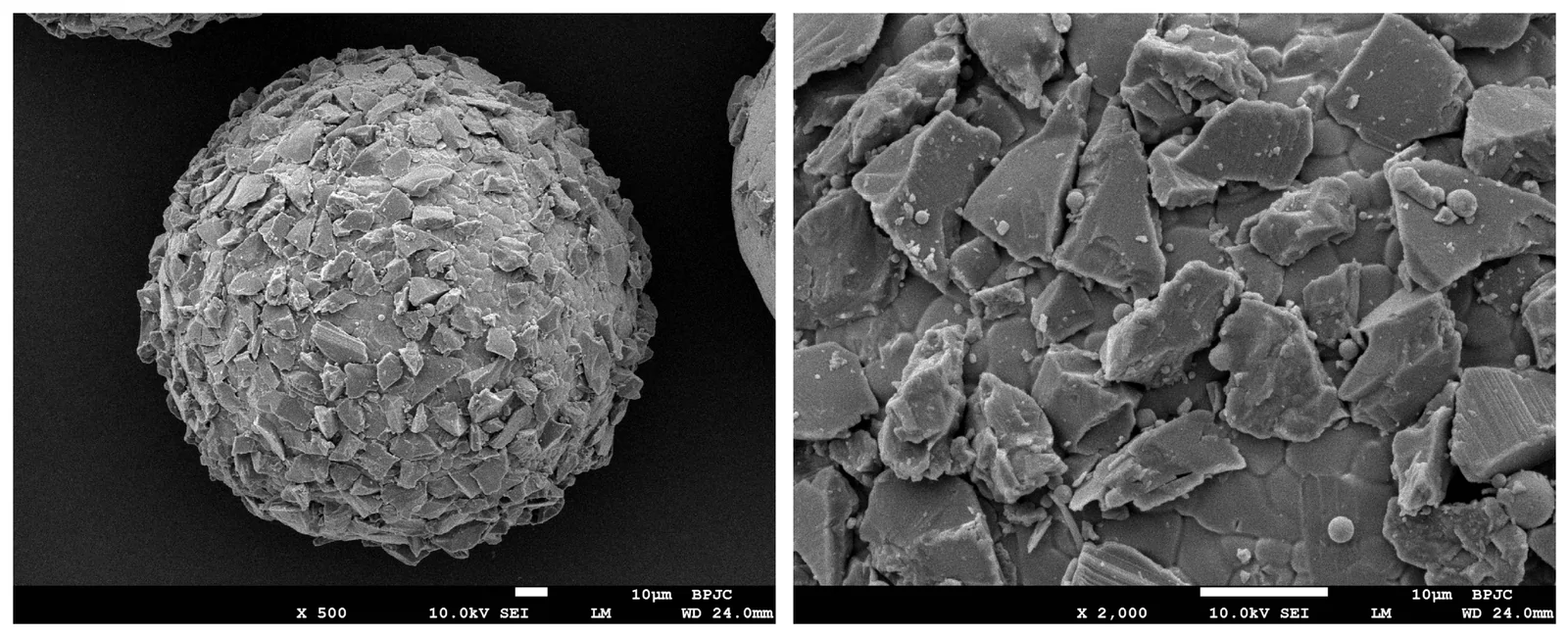
SEM images of iron-based diamond magnetic abrasives.

Figure 3 presents SEM micrographs of the prepared iron-based diamond magnetic abrasive powders. It can be clearly observed from the micrographs that the MAPs prepared by this method exhibit an ideal spherical morphology. The hard abrasive particles are predominantly distributed in the superficial layer of the metallic matrix, with their cutting edges protruding outward, and they are firmly bonded to the metallic matrix.

After preparation via plasma melting and centrifugal powder ejection rapid solidification, the spherical iron-based diamond magnetic abrasives were sieved using standard sieves to obtain the nominal size range of 100–160 μm for subsequent experiments. Considering the tube inner diameter of only 1.6 mm, particles significantly larger than 160 μm would hinder smooth injection and uniform distribution within the tube cavity, potentially causing clogging and inconsistent finishing; conversely, particles smaller than 100 μm exhibit insufficient indentation depth under the same magnetic field, resulting in low material removal efficiency. In addition, the selected size range (100–160 μm) is compatible with standard sieve mesh sizes (100 mesh to 160 mesh), facilitating convenient sieving classification and reproducible preparation.

## 3. Experimental Design

### 3.1. Equipment and Materials

**Figure 4:**
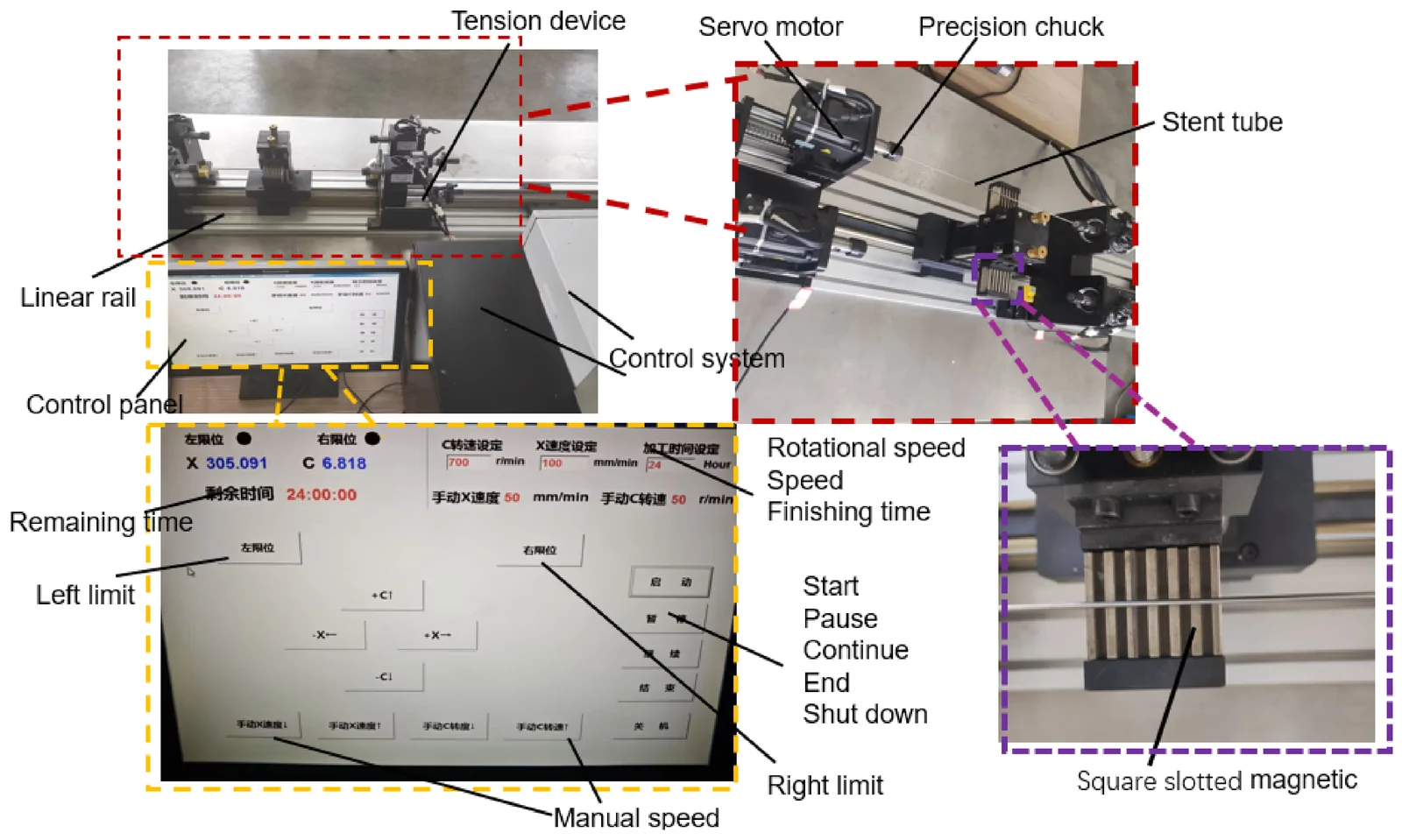
Magnetic abrasive finishing device for inner wall of cobalt–chromium alloy cardiovascular stent tube.

To address the polishing requirements of the inner wall of Co–Cr alloy vascular stent tubing, Figure 4 illustrates the developed magnetic finishing apparatus for the inner wall of ultra-fine and slender vascular stent tubing. The apparatus mainly consists of four subsystems: a tube rotation system, a magnetic field generation system, a transmission system and a control system. The tube rotation system employs an AC servo motor to drive a precision collet chuck, enabling reliable clamping and rotation of the workpiece. It is also equipped with a tensioning device, which allows adjustment of the machining stroke according to the tubing length and prevents bending of the tube, thereby accommodating the processing of cardiovascular stent tubing of varying lengths (e.g., up to 2 m). The magnetic field generation system adopts Nd–Fe–B permanent magnets as magnetic poles, which are fixed on the sliding platform of a synchronous belt linear module. The transmission system drives the synchronous belt via a stepper motor, which in turn drives the magnetic pole to perform axial reciprocating feed along the workpiece. The stroke is controlled by limit switches or proximity switches. The operation interface of the control system is programmed in Delphi, while the control program is developed in VC++, enabling the regulation of the servo motor speed and direction, the adjustment of pulse signals for the stepper motor, and the control of the magnetic pole feed rate. These subsystems work in a coordinated manner, allowing the magnetic abrasive particles to perform efficient polishing of the inner wall under the action of the magnetic field. Experimental results indicate that the developed apparatus can effectively perform magnetic finishing of the inner wall of Co–Cr alloy cardiovascular stent tubing, significantly improving the inner surface quality.

**Figure 5:**
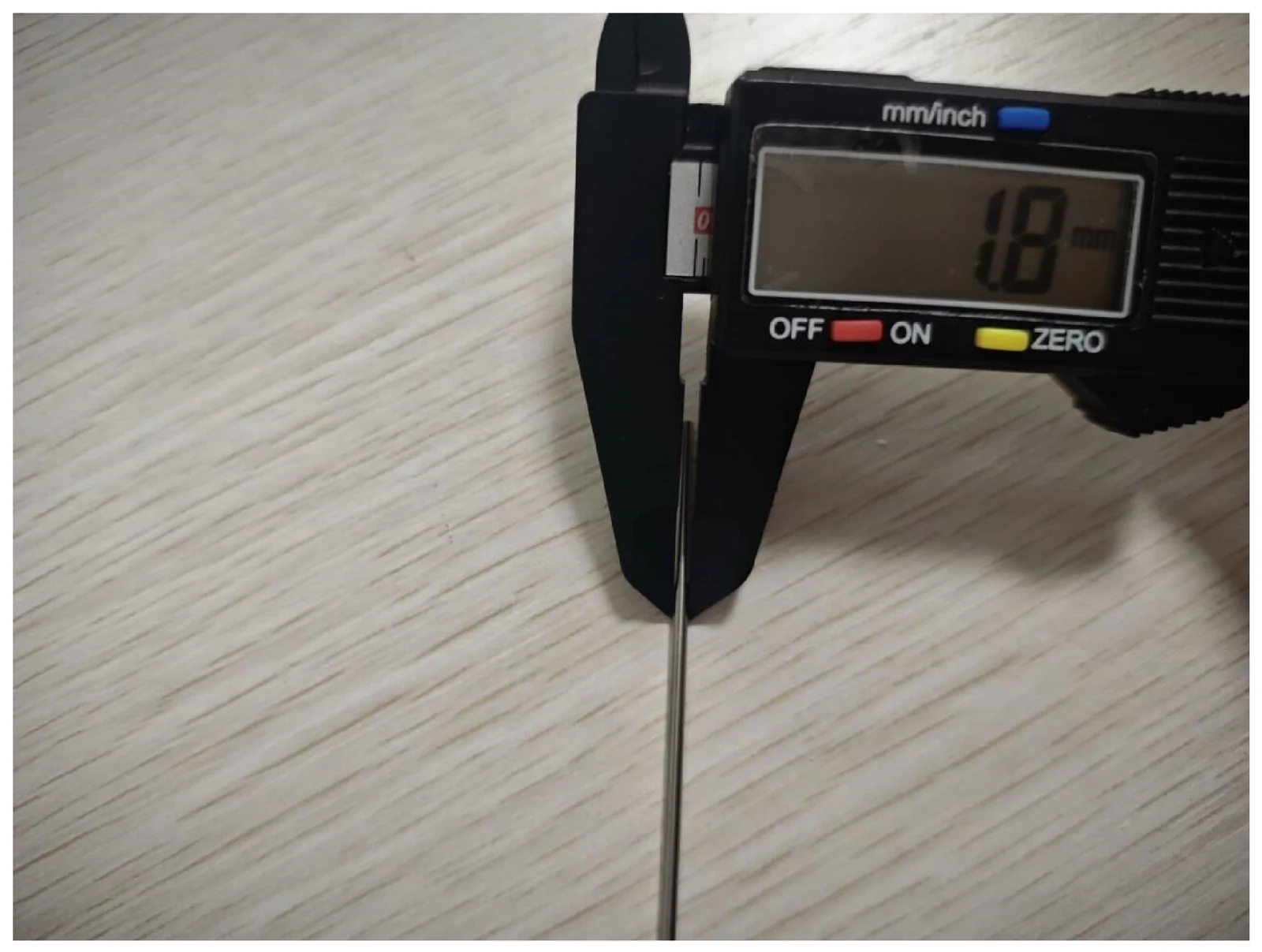
Photograph of L605 cobalt-chromium alloy vascular stent tube for testing.

The Co–Cr alloy vascular stent tubing used in the experiments is schematically illustrated in Figure 5. It has an outer diameter of 1.8 mm, an inner diameter of 1.6 mm and a length of 1800 mm. Its chemical composition and selected mechanical and physical properties are presented in Table 3 and Table 4 [23], respectively. Considering the considerable length of the Co–Cr alloy vascular stent tubing used in the experiments, each piece of tubing was cut into 600-mm-long sections for each experimental group to reduce the experimental cost.

### 3.2. Investigation of Surface Roughness in MAF of the Inner Wall of Co–Cr Alloy Vascular Stent Tubing

Before the experiment, the tube was cut into specimens of 600 mm in length. It should be noted that measurement of the inner wall roughness requires the tube to be axially sectioned, which is a destructive detection procedure. Consequently, it is impossible to measure the exact same position before and after machining on the same specimen. To address this limitation, a statistical comparison strategy was adopted in this study. Prior to machining, six measurement points were evenly distributed along the axial direction within the machining zone according to ISO 4288 [24], and the Ra value was measured at each point using a 3D digital microscope (DSX 1000, Olympus Corporation, Tokyo, Japan). At each point, multiple measurements were taken and averaged to obtain the roughness value for that point, and the arithmetic mean of these six points was taken as the initial surface roughness. The initial surface roughness Ra of the Co–Cr alloy cardiovascular stent tube inner wall was determined to be 0.587 μm. All roughness measurements were performed following ISO 4288 standards, with a cut-off wavelength (λc) of 0.8 mm and a sampling length of 0.8 mm. The evaluation length comprised 5 consecutive sampling lengths (4.0 mm in total). These parameters were selected to appropriately filter out waviness and form errors while capturing the relevant roughness features. After machining, another specimen from the same batch was processed under identical conditions, and the post-process Ra was obtained by following the same procedure within the same machining zone. The mean value of these post-process measurements was used to evaluate the finishing effect by comparison with the pre-process baseline.

For abrasive preparation, spherical iron-based diamond magnetic abrasives with different particle sizes were classified using standard sieves and weighed using a precision balance (accuracy 0.001 g). During machining, the weighed abrasives were first injected evenly into the tube cavity, followed by the oil-based grinding fluid. The grinding fluid serves a dual purpose: it facilitates the formation of a flexible magnetic brush by the abrasive particles under the magnetic field, and it provides lubrication and cooling to reduce thermal damage during processing.

After each set of experiments, the tube was immediately cleaned in an ultrasonic cleaner with anhydrous ethanol for 15 min to remove residual abrasives and oil contaminants, ensuring that the residues did not interfere with subsequent roughness measurements.

#### 3.2.1. Effect of Tube Rotational Speed on Surface Roughness

**Figure 6:**
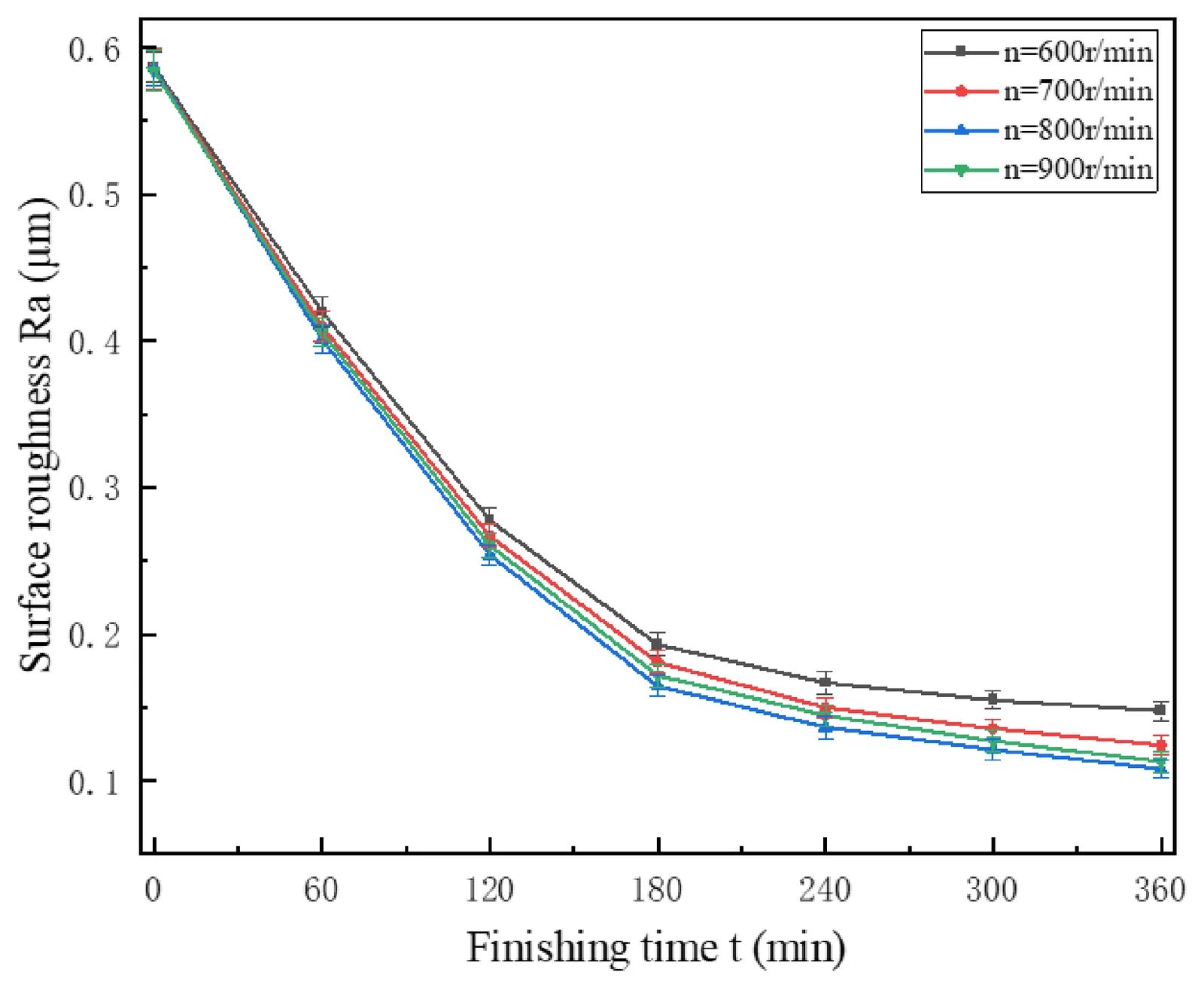
Effect of rotational speed on surface roughness.

Figure 6 shows the effect of tube rotational speed on surface roughness under the conditions specified in Table 5. The experimental results indicate that a favorable surface roughness was achieved at a tube rotational speed of 800 r·min^−1^. This is attributed to the fact that, at a constant magnetic pole feed rate, increasing the tube rotational speed prolongs the relative motion path of the magnetic abrasive brush with the inner wall per unit time, thereby increasing the frequency of interactions between the magnetic abrasive particles and the inner wall surface, and consequently enhancing the removal efficacy of the defect layer. However, excessive rotational speeds generate substantial centrifugal and frictional forces. When these forces exceed the magnetic field’s constraint capacity on the abrasive particles, the structure of the magnetic abrasive brush is disrupted, causing the abrasives to become discretely distributed and significantly weakening the finishing effect. Therefore, it can be concluded that a favorable surface roughness can only be achieved by appropriately increasing the tube rotational speed under conditions where the magnetic field forces and kinematic conditions are well matched.

#### 3.2.2. Effect of Magnetic Pole Feed Rate on Surface Roughness

**Figure 7:**
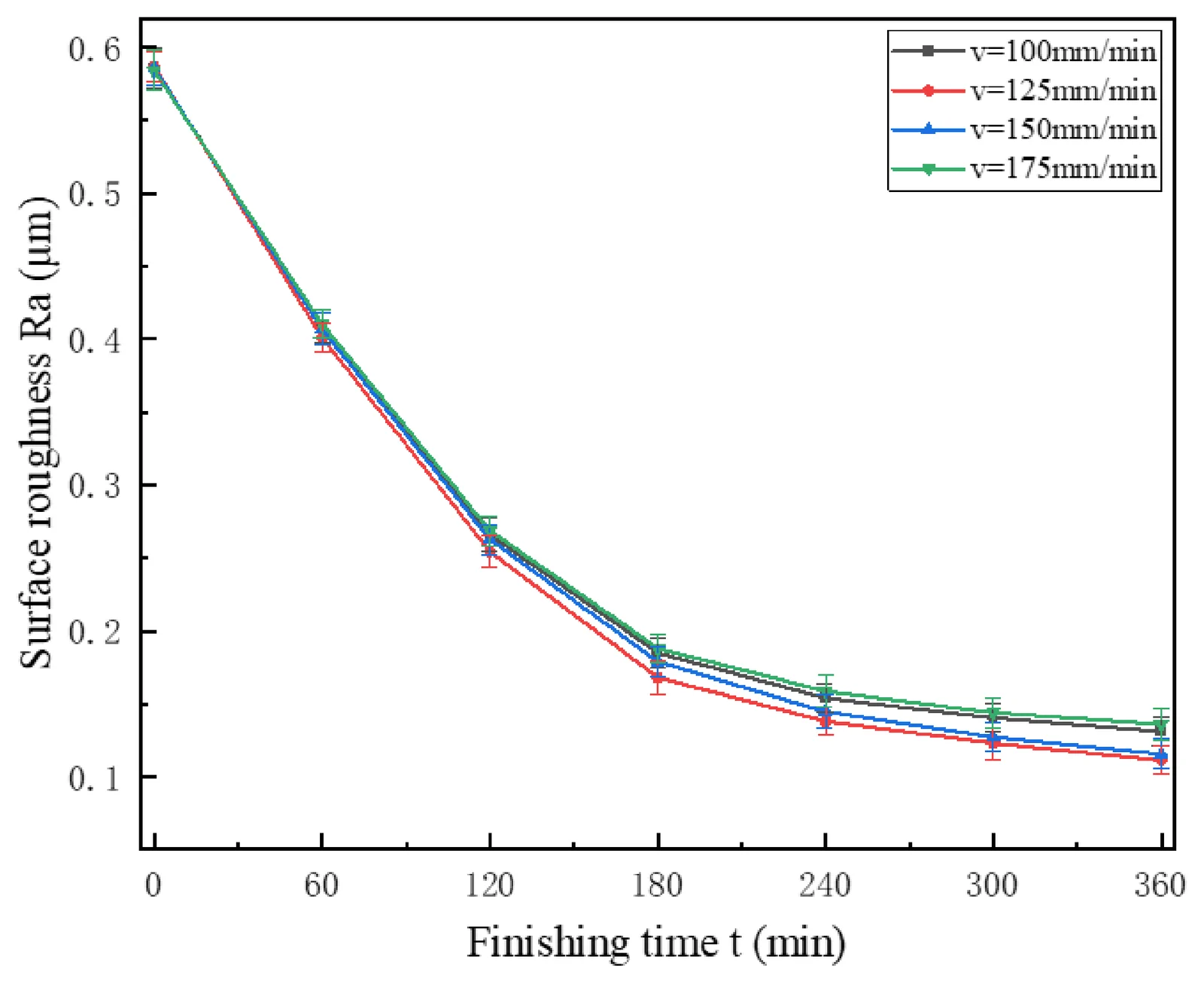
Effect of pole feeding speed on surface roughness.

Figure 7 shows the effect of magnetic pole feed rate on surface roughness under the conditions listed in Table 6 The experimental results indicate that a favorable surface roughness was achieved at a magnetic pole feed rate of 125 mm·min^−1^. This is attributed to the fact that, at a constant tube rotational speed, a lower magnetic pole feed rate results in a denser motion trajectory of the magnetic abrasive brush on the inner wall surface, significantly increasing the number of repeated actions on the same region and thereby rendering the material removal process more uniform. However, the feed rate should not be excessively low, as an overly slow magnetic pole feed rate prolongs the path length of repeated processing, leading to excessive material removal induced by the magnetic abrasive particles on the inner wall. Conversely, an excessively high feed rate makes it difficult for the abrasive particles to synchronize with the motion rhythm of the magnetic pole, causing some particles to deviate from the effective finishing area. This reduces the actual number of abrasive particles participating in the finishing process, thereby weakening the overall finishing effect.

#### 3.2.3. Effect of Abrasive Particle Size on Surface Roughness

**Figure 8:**
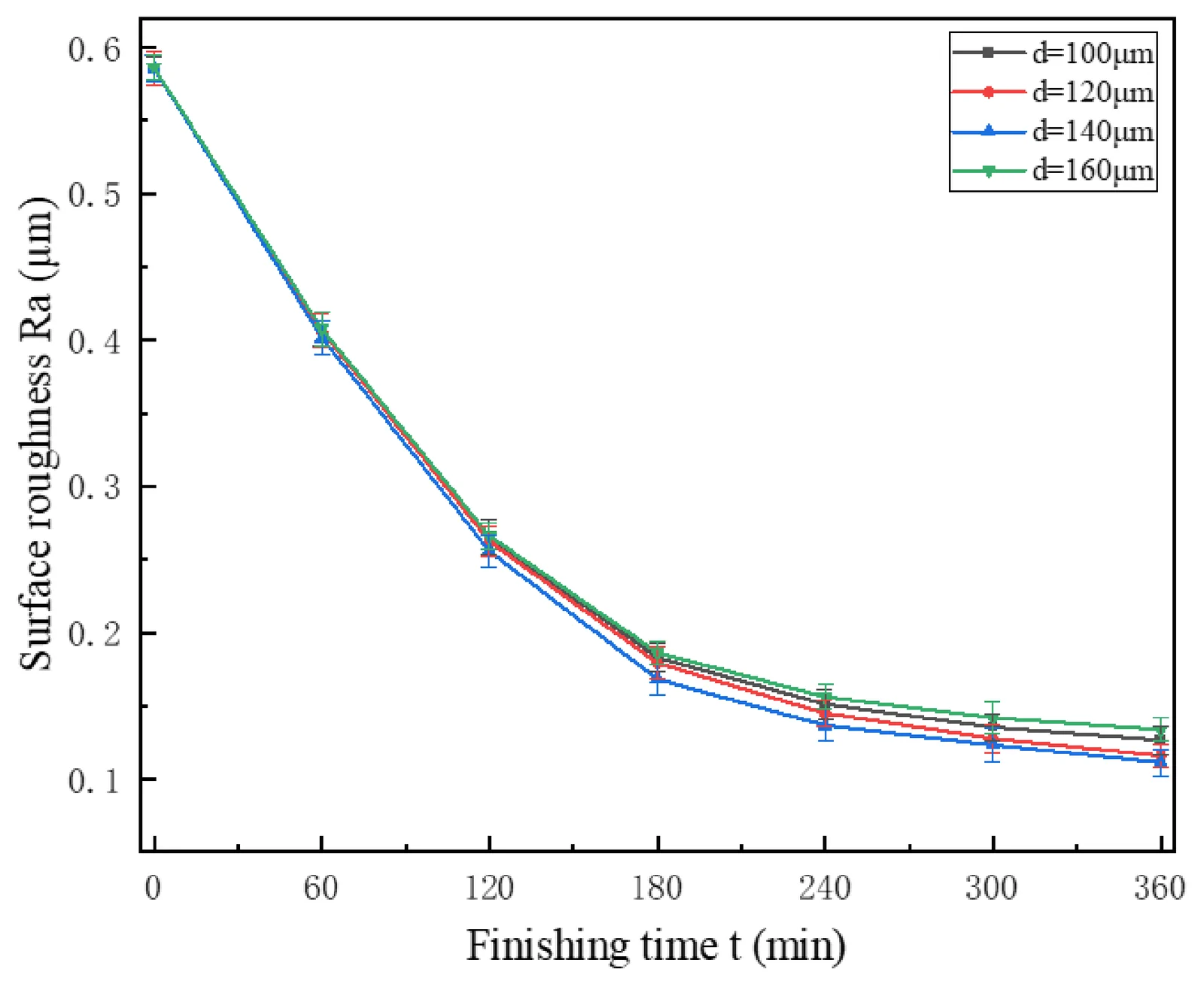
Effect of abrasive particle size on surface roughness.

Figure 8 shows the effect of abrasive particle size on surface roughness under the conditions listed in Table 7. The experimental results indicate that a favorable surface roughness was achieved at an abrasive particle size of 140 µm. This is attributed to the fact that, under identical experimental conditions, a larger abrasive particle size generates a greater normal pressure on the inner wall of the tubing, resulting in a deeper indentation depth, which enables more effective removal of the defect layer from the inner wall surface. However, after the defect layer has been removed, the excessive normal pressure may cause the abrasive particles to produce new scratches on the inner wall, thereby compromising the surface quality. Conversely, if the abrasive particle size is too small, the indentation depth becomes insufficient, leading to incomplete removal of the defect layer and consequently degrading the finishing performance.

#### 3.2.4. Effect of Working Gap on Surface Roughness

**Figure 9:**
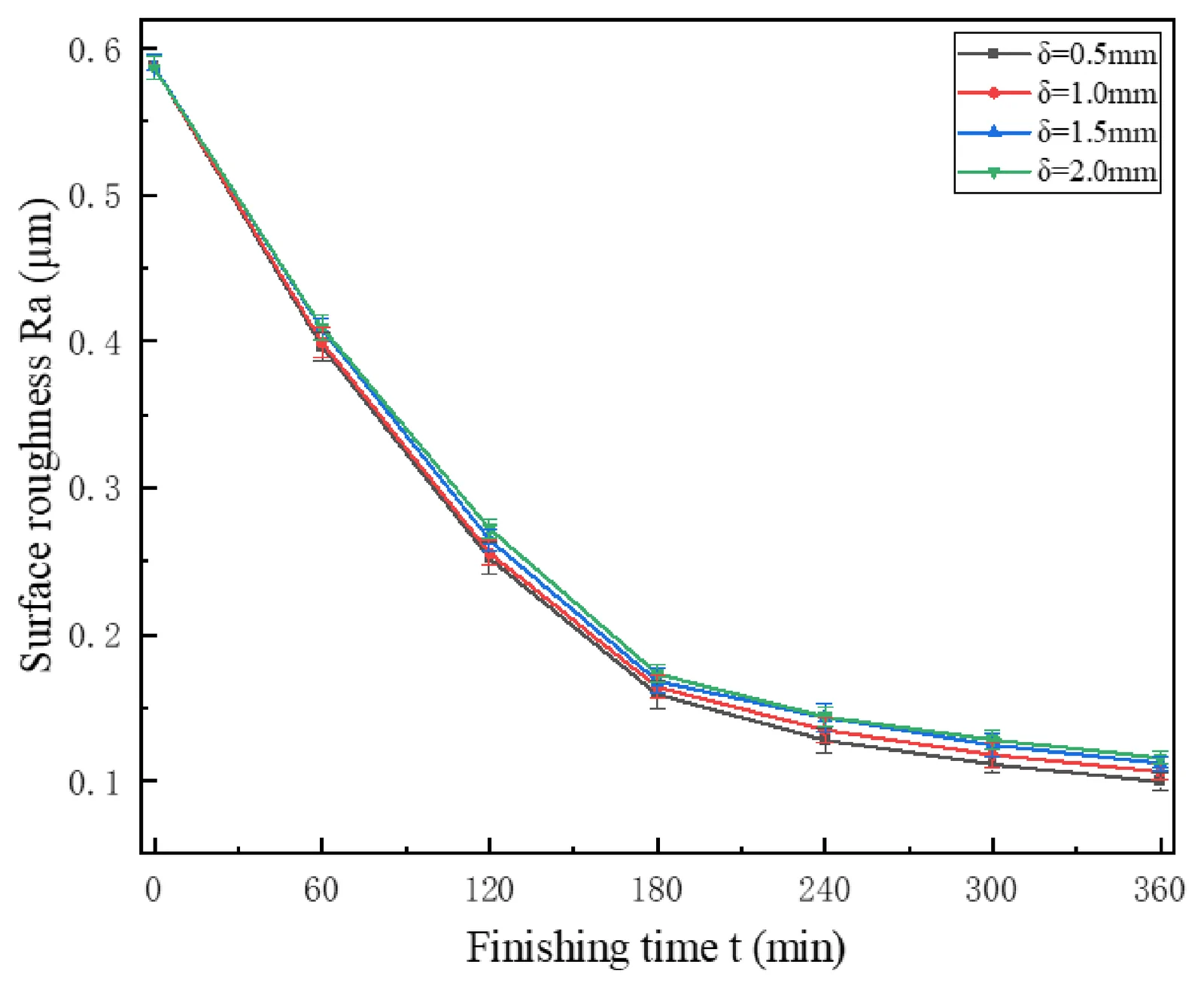
Effect of machining gap on surface roughness.

Figure 9 shows the effect of working gap on surface roughness under the conditions listed in Table 8. The experimental results indicate that a favorable surface roughness was achieved at a working gap of 0.5 mm. This is attributed to the fact that a smaller working gap results in higher magnetic flux density within the machining area, generating stronger magnetic field forces that enable the hard abrasive particles to embed more effectively into the defect layer. As the working gap gradually increases, the magnetic field lines become more dispersed, leading to a substantial decrease in the actual magnetic flux density within the machining area. Consequently, the hard abrasive particles are unable to embed effectively into the defect layer, resulting in a marked deterioration of the finishing performance.

## 4. PSO-SVM-Based Surface Roughness Prediction Model

### 4.1. Orthogonal Experimental Design and Results

Orthogonal experimental design is founded on mathematical principles, enabling the selection of representative factors and levels from a large number of experimental variables. This approach ensures reliable experimental outcomes while substantially reducing the number of trials, and it has been widely applied in experiments involving multiple factors and multiple levels [25,26]. To qualitatively analyze the influence of various process parameters on surface roughness Ra and to identify the parameter combination that minimizes surface roughness under given conditions, an L_16_(4^4^) orthogonal array was designed. MAF experiments were conducted on the inner wall of Co–Cr alloy vascular stent tubing with an inner diameter of 1.6 mm and an outer diameter of 1.8 mm using the developed experimental setup, with a finishing duration of 360 min. The factor levels and the orthogonal experimental results are presented in Table 9 and Table 10, respectively.

Range analysis is a commonly used analytical method in orthogonal experiments. The range R_j_ serves as a key indicator for measuring the strength of factor effects, characterizing the maximum fluctuation amplitude of a specific factor on the observed results under different levels [27]. A larger R_j_ value indicates a stronger influence of the factor’s level changes on the surface roughness Ra, and a more prominent dominant role of this factor in the process system. According to the range analysis results presented in Table 11, the order of significance of the four process parameters on surface roughness Ra is: tube rotational speed > working gap > magnetic pole feed rate > abrasive particle size.

### 4.2. Support Vector Machine (SVM)

The magnetic abrasive finishing (MAF) process inherently exhibits a certain degree of randomness and volatility. To address this issue, support vector machine (SVM) was employed to construct a prediction model for surface roughness Ra.

The L_16_(4^4^) orthogonal experimental design yielded a total of 16 sets of data (Table 10, which were used as the sample set for model construction. The sample set was randomly shuffled and then partitioned into a training set of 12 samples and a test set of 4 samples, with a fixed random seed of 42 to ensure reproducibility. The four input variables of the prediction model were tube rotational speed, magnetic pole feed rate, abrasive particle size, and working gap, and the output variable was surface roughness Ra. All data were normalized prior to model training to accelerate convergence and eliminate the influence of dimensional differences among variables.

Given a training dataset consisting of *n* samples, denoted as ($x_{i}$,$y_{i}$), where i = 1, 2, 3, …, *n*, with $x_{i}\in R^{n}$ representing the input data and $R^{n}$ denoting the n-dimensional vector space, the target values $y_{i}$ were normalized to the interval [−1, 1] using Equation (1).

(1) $$y=\frac{x-x_{\min}}{x_{\max}-x_{\min}}$$

where $x_{max}$ and $x_{min}$ denote the maximum and minimum values of the input data, respectively. The purpose of data normalization is to reduce the magnitude discrepancies among attributes within the input vector. Excessive magnitude differences may adversely affect the final prediction results and potentially distort the predicted outcomes [28].

The classification philosophy of SVM assumes that the data are linearly separable, meaning that there exists a hyperplane that can separate data points of one class from all data points of the other class, from which the desired optimal solution can be derived [29]. The definition of the separating hyperplane f(x)=0 is given by Equation (2).

(2) $$f\left(x\right)=\omega x+b=0$$

where ω is the normal vector of the hyperplane, determining its orientation, and b denotes the bias, which determines the offset of the hyperplane.

Meanwhile, the regression function of the nonlinear prediction model was constructed, as shown in Equation (3).

(3) $$f\left(x_{i}\right)=sign\left(\omega x_{i}+b\right)$$

The objective of SVM is to find a hyperplane that maximizes the margin between classes. The margin is defined as the distance from the hyperplane to the nearest data points, which are referred to as support vectors. These support vectors satisfy the equation given in Equation (4).

(4) $$\omega x_{i}+b=\pm 1$$

In this case, the problem of solving for the optimal hyperplane in SVM can be transformed into an optimization problem of maximizing the margin. To better address this optimization problem, the Lagrange function was constructed, as shown in Equation (5).

(5) $$L\left(\omega ,b,\alpha \right)=\frac{1}{2}\|\omega {\|}^{2}-\sum_{i=1}^{n}\alpha_{i}\left[y_{i}\left(\omega x_{i}+b\right)-1\right]$$

Then, the dual transformation was performed, as shown in Equation (6).

(6) $$\left\{\begin{array}{l} \underset{\alpha }{\max}\sum_{i=1}^{n}\alpha_{i}-\frac{1}{2}\sum_{i=1}^{n}\sum_{j=1}^{n}\alpha_{i}\alpha_{j}y_{i}y_{j}x_{i}x_{j}\\ s.t.\sum_{i=1}^{n}\alpha_{i}y_{i}=0,\alpha_{i}\geq 0 \end{array}\right.$$

Subsequently, the optimal solution of ω and b was computed by optimizing $\alpha_{i}$.

(7) $$\omega =\sum_{i=1}^{n}\alpha_{i}x_{i}y_{i}$$

(8) $$b=y_{i}-\sum_{j=1}^{n}\alpha_{j}y_{j}x_{j}x_{i}$$

For nonlinearly separable data, a kernel function can be introduced to achieve linear separability. When dealing with nonlinear problems, the radial basis function (RBF) is typically selected as the kernel function to improve fitting accuracy and reduce prediction error, as shown in Equation (9).

(9) $$K\left(x_{i},x_{j}\right)=\exp\left(\frac{\|x_{i}-x_{j}{\|}^{2}}{2\sigma^{2}}\right)$$

where σ is the radius parameter of the kernel function, which determines the radial range of the kernel function.

Based on the above analysis, the surface roughness prediction model based on SVM was finally obtained, and the regression function is given by Equation (10).

(10) $$f\left(x\right)=sign\left(\sum_{i=1}^{n}\alpha_{i}y_{i}K\left(x_{i},x_{j}\right)+b\right)$$

### 4.3. Construction of PSO-SVM Surface Roughness Prediction Model

The performance of SVM largely depends on the selection of its key hyperparameters, particularly the kernel parameter γ and the penalty parameter C, The parameter γ controls the degree of nonlinearity of the model, while the parameter C governs the trade-off between model complexity and overfitting. However, manual tuning of these two parameters is time-consuming and requires extensive experimentation. Therefore, an effective method is needed to identify the optimal parameter combination. As a global optimization algorithm, particle swarm optimization (PSO) is capable of searching for the global optimum by simulating the cooperative behavior of a particle swarm within the search space. The fundamental principle of PSO is that particles iteratively update their positions and velocities to pursue the optimal solution of the target parameters.

In the PSO algorithm, each particle corresponds to a candidate solution. For the SVM parameter optimization problem, a particle represents a specific combination of SVM hyperparameters, such as the penalty parameter C and the RBF kernel parameter γ. Assuming a two-dimensional search space, the current position of each particle can be expressed as:

(11) $$x_{i}=\left[C_{i},\gamma_{i}\right]$$

The particle swarm continuously updates its velocity and position through iteration, progressively approaching the optimal solution. The velocity update formula for the particle swarm is given by Equation (12).

(12) $$v_{i}\left(k+1\right)=\omega v_{i}\left(k\right)+c_{1}\gamma_{1}\left(p_{i}^{best}-x_{i}\left(k\right)\right)+c_{2}\gamma_{2}\left(g^{best}-x_{i}\left(k\right)\right)$$

where ω is the inertia weight factor, $c_{1},c_{2}$ are positive acceleration coefficients, $p_{i}^{best}$ denotes the historical best position of the particle and $g^{best}$ represents the global best position found by the entire swarm. The variables $\gamma_{1}$ and $\gamma_{2}$ are random numbers uniformly distributed in the range [0, 1].

Meanwhile, the particle position update formula is given as follows:

(13) $$x_{i}\left(k+1\right)=x_{i}\left(k\right)+v_{i}\left(k+1\right)$$

### 4.4. Prediction Model Analysis

The prediction accuracy and predictive performance of the PSO-SVM model were evaluated using the coefficient of determination (R^2^), root-mean-square error (RMSE) and mean absolute percentage error (MAPE) calculated from the test samples. The corresponding formulas are given as follows:

(14) $$R^{2}=1-\frac{\sum_{i=1}^{4}{\left(y_{Ra}-y_{i}\right)}^{2}}{\sum_{i=1}^{4}{\left(y_{i}-\bar{y}\right)}^{2}}$$

(15) $$RMSE=\sqrt{\frac{\sum_{i=1}^{4}{\left(y_{i}-y_{Ra}\right)}^{2}}{4}}$$

(16) $$MAPE=\frac{\sum_{i=1}^{4}\left|\frac{y_{i}-y_{Ra}}{y_{i}}\right|}{4}\times 100\%$$

where $y_{i}$ is the actual value and $y_{Ra}$ is the predicted value. The coefficient of determination $R^{2}$ is employed to evaluate the goodness of fit of the regression model to the sample space, characterizing the proportion of the total variance in the dependent variable that is explained by the model. RMSE is used to measure the quality of the model fit, while MAPE is adopted to assess the volatility of the predicted data.

**Figure 10:**
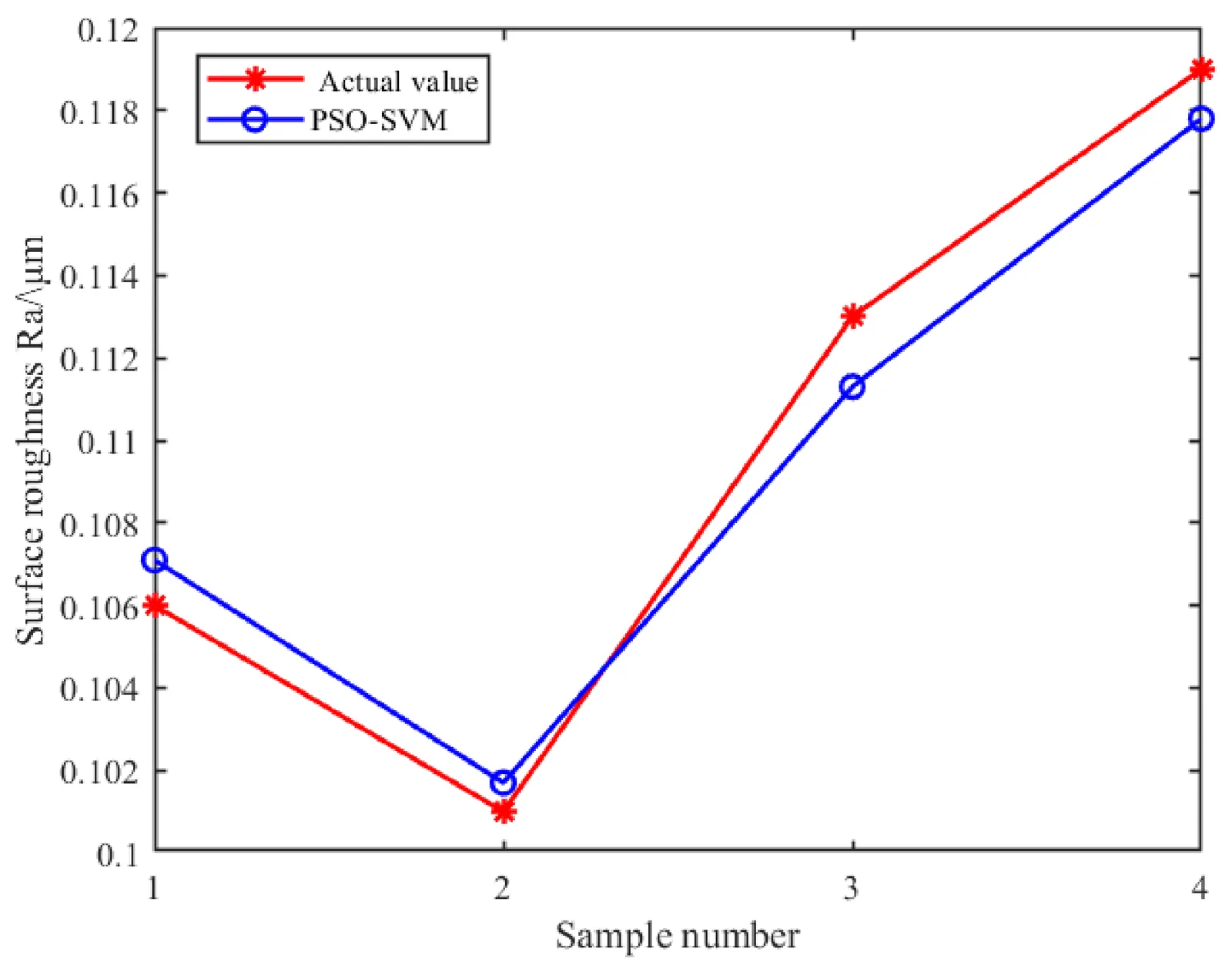
Predicted curve and actual curve of PSO-SVM model.

The PSO-SVM model was constructed using MATLAB (R2024a) software. The prediction results of the test samples are presented in Figure 10, the corresponding prediction errors are listed in Table 12 and the calculated values of $R^{2}$, RMSE and MAPE are summarized in Table 13.

## 5. Optimization of Process Parameters

### 5.1. Parameter Settings and Optimization Results

**Figure 11:**
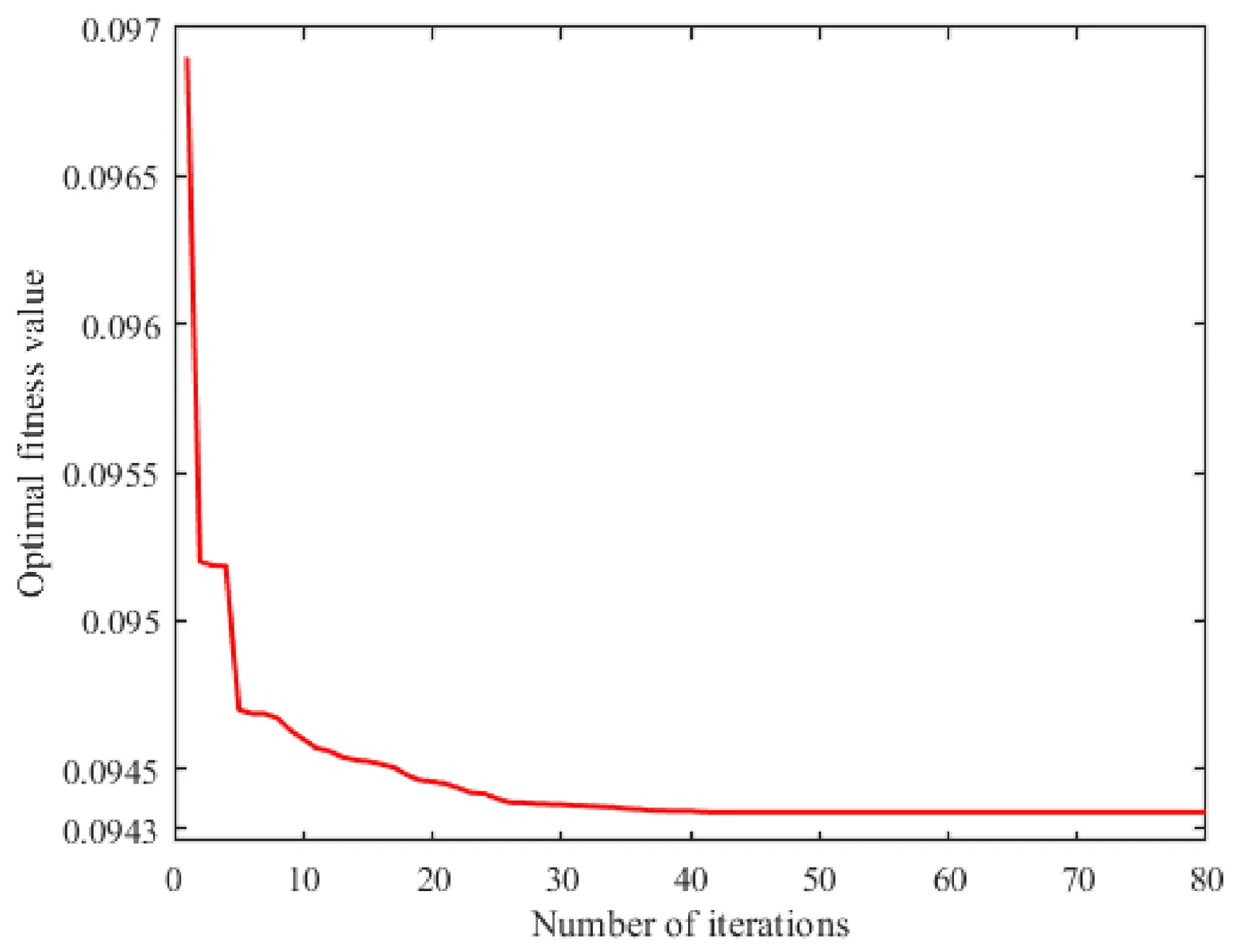
The curve of fitness value.

To obtain the optimal process parameter combination for better surface quality, the PSO-SVM model was employed for process parameter optimization. The optimization ranges of the process parameters are presented in Table 14. After 80 iterations, the optimal process parameter combination was obtained as: (*n*, v, d, δ) = (832.6384 r·min^−1^, 129.6784 mm·min^−1^, 132.4185 µm, 0.5324 mm), with a corresponding surface roughness Ra of 0.09435 µm, The fitness evolution curve is shown in Figure 11.

### 5.2. Experimental Verification

Considering the inherent limitations of the existing experimental equipment, the optimized process parameters were appropriately fine-tuned within the maximum feasible range of the apparatus. The fine-tuned parameter combination was set as: (*n*, v, d, δ) = (833 r·min^−1^, 130 mm·min^−1^, 132 µm, 0.5 mm). Apart from the parameter adjustments, all other experimental conditions and data acquisition methods remained unchanged. Under these settings, three parallel experiments were conducted, and the experimental results are presented in Table 15.

**Figure 12:**
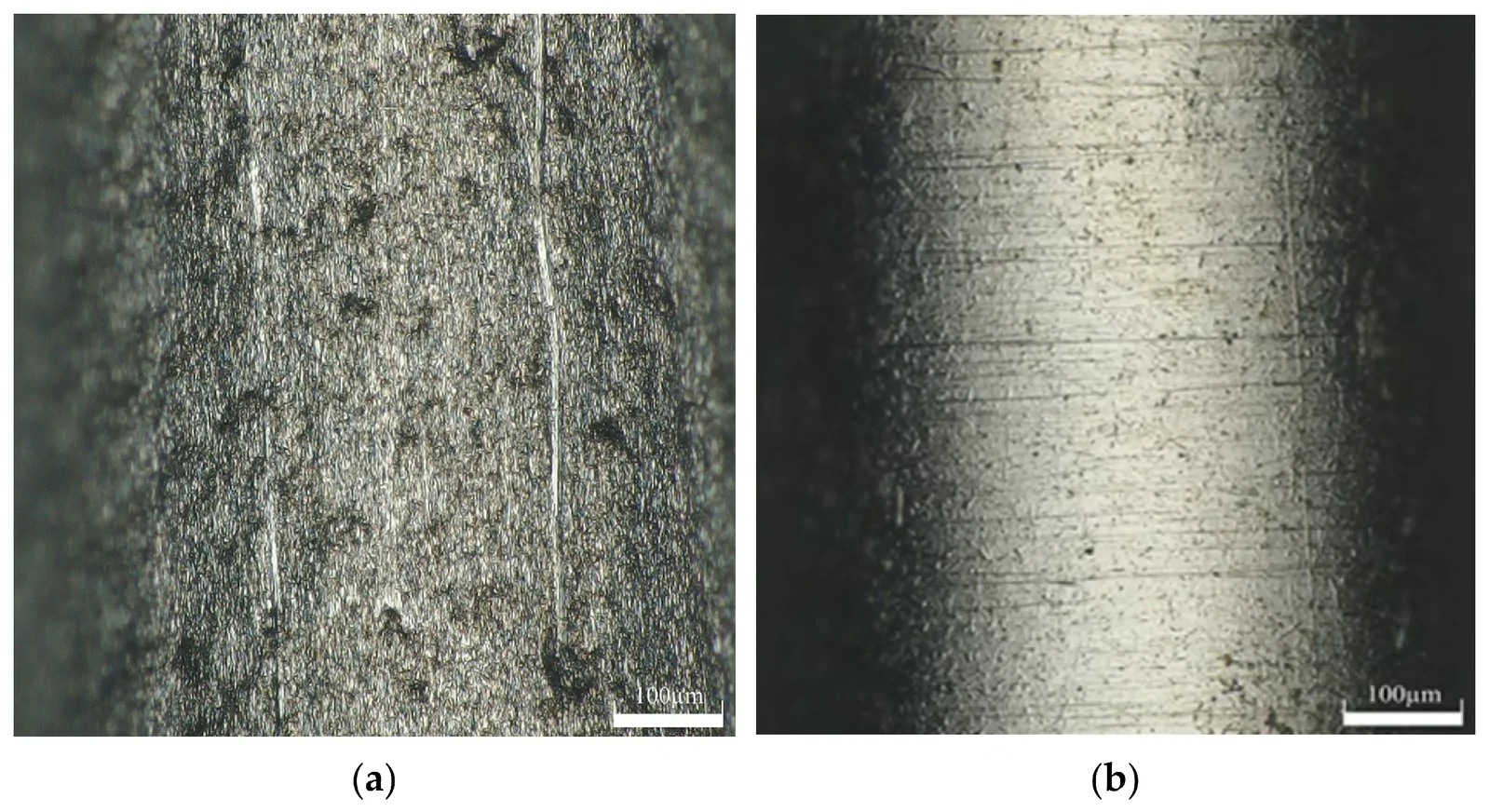
2D surface topography of the inner wall of Co–Cr alloy vascular stent tubing (**a**) before MAF and (**b**) after MAF.

**Figure 13:**
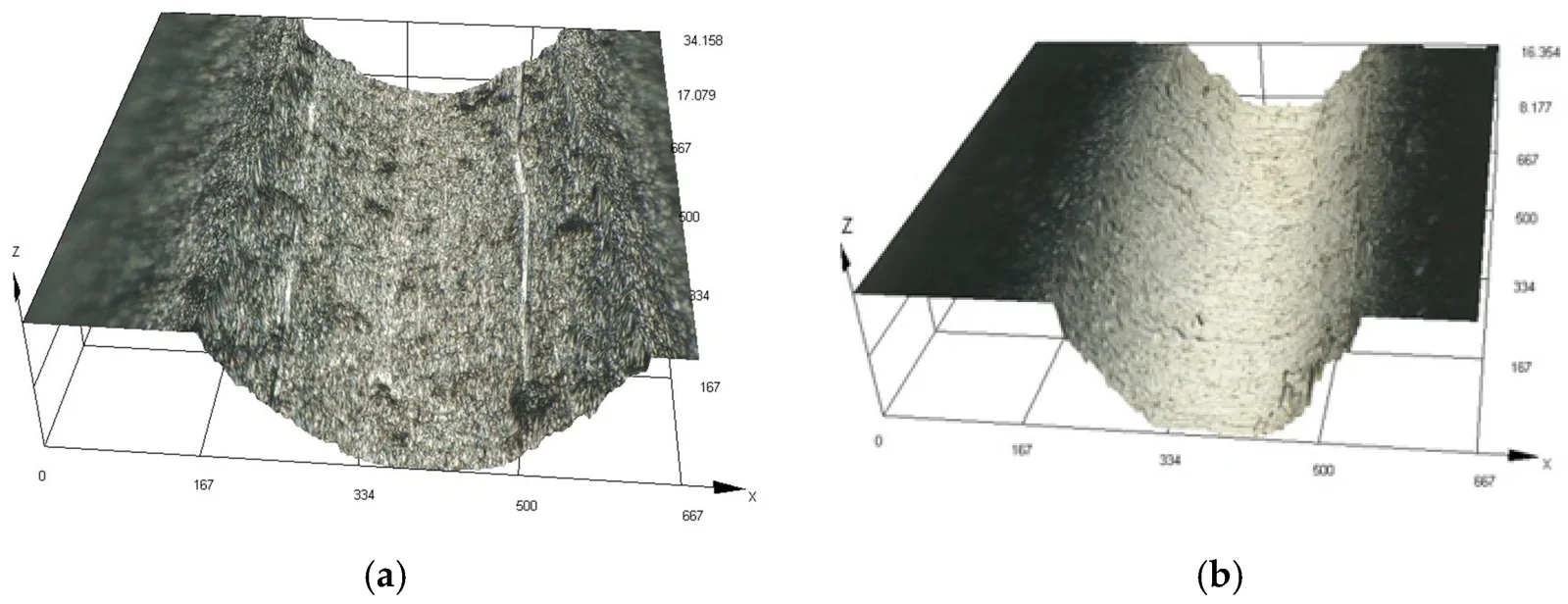
3D surface topography of the inner wall of Co–Cr alloy vascular stent tubing (**a**) before MAF and (**b**) after MAF.

**Figure 14:**
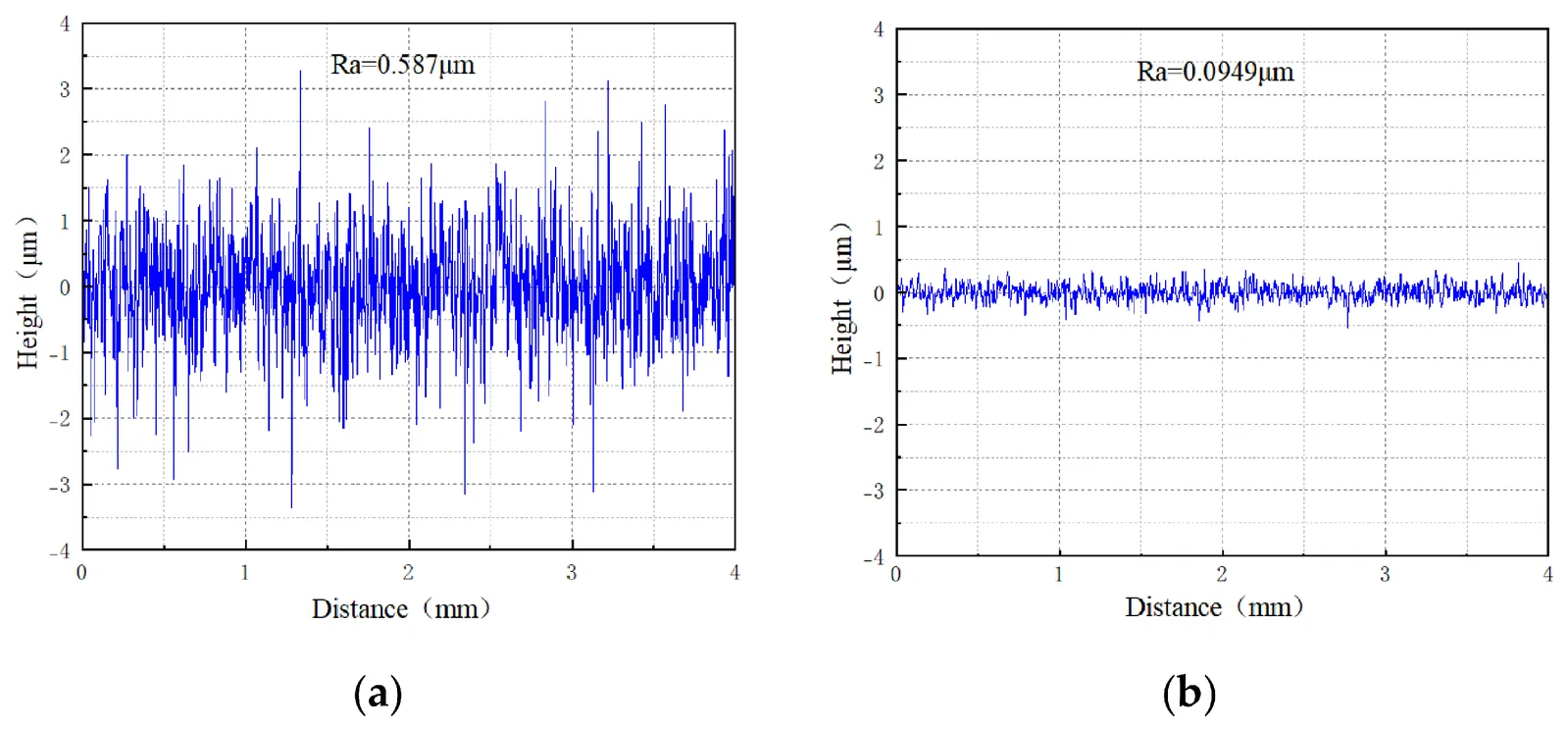
2D roughness profiles of the inner wall surface: (**a**) before MAF; (**b**) after MAF.

The inner surface morphology of the Co–Cr alloy vascular stent tubing was observed and the surface roughness was measured using a 3D digital microscope (DSX 1000, Olympus Corporation, Tokyo, Japan). Figure 12a and Figure 13a show the 2D and 3D surface morphologies of the inner wall of the Co–Cr alloy vascular stent tubing before processing, respectively. It can be clearly observed from the figures that the inner wall surface exhibited obvious defects prior to MAF, including protrusions, pits and craters, with a measured surface roughness value of 0.587 μm, as shown in Figure 14a. Figure 12b and Figure 13b show the 2D and 3D surface morphologies after processing under the optimized process parameters, respectively. It can be clearly observed from the figures that the number of scratches on the processed surface was significantly reduced, and defects such as wrinkles and craters were effectively eliminated. The surface became smoother and more uniform, indicating a substantial improvement in surface quality. The measured surface roughness was 0.0949 μm, as shown in Figure 14b.

## 6. Conclusions

This study systematically investigated the magnetic abrasive finishing (MAF) process for the inner wall of Co–Cr alloy vascular stent tubing, and successfully established a PSO-SVM-based prediction and optimization framework for surface roughness control. The main findings are summarized as follows:

(1) Iron-based diamond magnetic abrasive powders (MAPs) with ideal spherical morphology were successfully prepared via plasma melting, centrifugal spraying and rapid solidification. The prepared MAPs exhibit favorable cutting performance and can be effectively employed for magnetic abrasive finishing (MAF) of the inner wall of cardiovascular stent tubing.
(2) The effects of tube rotational speed, magnetic pole feed rate, abrasive particle size and working gap on the surface roughness Ra of the inner wall of Co–Cr alloy cardiovascular stent tubing in magnetic abrasive finishing (MAF) were systematically investigated.
(3) Based on the orthogonal experimental results, an SVM prediction model was constructed with tube rotational speed, magnetic pole feed rate, working gap and abrasive particle size as input variables and surface roughness Ra as the output response. The two key hyperparameters of SVM were optimized using the particle swarm optimization (PSO) algorithm, thereby establishing a PSO-SVM prediction model with a coefficient of determination (R^2^) of 0.96771, a root-mean-square error (RMSE) of 0.0012756 μm, and a mean absolute percentage error (MAPE) of 1.061%, enabling accurate prediction of surface roughness.
(4) The PSO algorithm was further employed to optimize the process parameters using the constructed PSO-SVM prediction model. When the tube rotational speed was 832.6384 r·min^−1^, the magnetic pole feed rate was 129.6784 mm·min^−1^, the working gap was 0.5324 mm, and the abrasive particle size was 132.4185 µm, the minimum surface roughness Ra of 0.0949 μm was achieved.
(5) Despite the promising results obtained in this study, several limitations should be acknowledged. First, due to the destructive nature of the inner wall roughness measurement, pre- and post-machining comparisons at identical positions could not be achieved on the same specimen. Second, this study only employed Ra as the surface quality indicator, without systematic evaluation of Rq and Rz parameters and their effects on stent fatigue performance and hemocompatibility. In addition, the control precision of the experimental equipment still needs to be further improved to meet the requirements for finer adjustment of process parameters. Future work will focus on addressing these limitations to further advance the application of MAF technology in cardiovascular stent manufacturing, including systematic characterization of Rq/Rz and their correlations with stent service performance.

## Figures and Tables

**Table 1:** Parameter settings of the feeding device.

Parameter	Value
Gas pressure (MPa)	0.52
Disc speed (r/min)	3
Feeding rate (L/h)	300

**Table 2:** Parameters of plasma generation device.

Parameter	Value
Hydrogen pressure (MPa)	0.61
Argon pressure (MPa)	0.57
Hydrogen flow rate (L/h)	87
Argon flow rate (L/h)	900
Actual voltage (V)	52.61
Actual current (A)	550

**Table 3:** Composition of L605 Co–Cr alloy stent tube.

Element	Cr	W	Ni	Fe	C	Si	Mn	Co
MASS Fraction w/%	19~21	14~16	9~11	≤3	≤0.15	≤1	≤2	Bal.

**Table 4:** Mechanical properties of L605 Co–Cr alloy stent tube.

Performance Indicators	Density (g·cm^−3^)	Elastic Modulus (GPa)	Tensile Strength (MPa)	Yield Strength (MPa)	Elongation (%)	Elastic Range (%)
Value	9.10	243	820~1200	420~600	35~55	0.16~0.32

**Table 5:** Single-factor experimental parameters.

Parameter	Value
Finishing time (min)	0, 60, 120, 180, 240, 300, 360
Pipe rotational speed *n* (r·min^−1^)	600, 700, 800, 900
Pole entry velocity v (mm·min^−1^)	150
A abrasive size d (µm)	120
Working gap δ (mm)	1

**Table 6:** Single-factor experimental parameters.

Parameter	Value
Finishing time (min)	0, 60, 120, 180, 240, 300, 360
Pole entry velocity v (mm·min^−1^)	100, 125, 150, 175
Pipe rotational speed *n* (r·min^−1^)	800
A abrasive size d (µm)	120
Working gap δ (mm)	1

**Table 7:** Single-factor experimental parameters.

Parameter	Value
Finishing time (min)	0, 60, 120, 180, 240, 300, 360
A abrasive size d (µm)	100, 120, 140, 160
Pipe rotational speed *n* (r·min^−1^)	800
Pole entry velocity v (mm·min^−1^)	125
Working gap δ (mm)	1

**Table 8:** Single-factor experimental parameters.

Parameter	Value
Finishing time (min)	0, 60, 120, 180, 240, 300, 360
Working gap δ (mm)	0.5, 1.0, 1.5, 2.0
Pipe rotational speed *n* (r·min^−1^)	800
Pole entry velocity v (mm·min^−1^)	125
A abrasive size d (µm)	140

**Table 9:** Factor level table.

Level	Pipe Rotational Speed *n* (r·min^−1^)	Pole Entry Velocity v (mm·min^−1^)	A Abrasive Size d (µm)	Working Gap δ (mm)
1	600	100	100	0.5
2	700	125	120	1.0
3	800	150	140	1.5
4	900	175	160	2.0

**Table 10:** Results of orthogonal experiments.

Level	Pipe Rotational Speed *n* (r·min^−1^)	Pole Entry Velocity v (mm·min^−1^)	A Abrasive Size d (µm)	Working Gap δ (mm)	Surface Roughness Ra (µm)
1	600	100	100	0.5	0.121
2	600	125	120	1.0	0.156
3	600	150	140	1.5	0.152
4	600	175	160	2.0	0.162
5	700	100	120	1.5	0.131
6	700	125	100	2.0	0.127
7	700	150	160	0.5	0.119
8	700	175	140	1.0	0.124
9	800	100	140	2.0	0.115
10	800	125	160	1.5	0.109
11	800	150	100	1.0	0.106
12	800	175	120	0.5	0.098
13	900	100	160	1.0	0.104
14	900	125	140	0.5	0.101
15	900	150	120	2.0	0.113
16	900	175	100	1.5	0.119

**Table 11:** Range analysis results.

	Pipe Rotational Speed *n* (r·min^−1^)	Pole Entry Velocity v (mm·min^−1^)	A Abrasive Size d (µm)	Working Gap δ (mm)
${\bar{K}}_{1}$	0.148	0.118	0.118	0.110
${\bar{K}}_{2}$	0.125	0.123	0.125	0.122
${\bar{K}}_{3}$	0.107	0.122	0.123	0.128
${\bar{K}}_{4}$	0.109	0.126	0.123	0.129
R_j_	0.041	0.008	0.007	0.019

**Table 12:** Comparison between experimental and predicted results.

Sample Number	Actual Surface Roughness Ra (µm)	Prediction Value Ra (µm)	Relative Error (%)
1	0.106	0.1071	1.04
2	0.101	0.1017	0.69
3	0.113	0.1113	1.50
4	0.119	0.1178	1.01

**Table 13:** Calculation results.

Parameter Name	Data Value
R^2^	0.96771
RMSE	0.0012756
MAPE	1.061%

**Table 14:** Optimization range of process parameters.

Object	Range
Pipe rotational speed *n* (r·min^−1^)	[600, 900]
Pole entry velocity v (mm·min^−1^)	[100, 175]
A abrasive size d (µm)	[100, 160]
Working gap δ (mm)	[0.5, 2.0]

**Table 15:** Optimization test verification results.

Sample Number	Actual Surface Roughness Ra/µm	Prediction Value Ra/µm	Relative Error/%
1	0.0957	0.09435	1.41
2	0.0951	0.09435	0.79
3	0.0949	0.09435	0.58

## Data Availability

The original contributions presented in this study are included in the article. Further inquiries can be directed to the corresponding author.

## References

[1] Antza C., Gallo A., Boutari C., Ershova A., Gurses K.M., Lewek J., Mirmaksudov M., Silbernagel G., Sandstedt J., Lebedeva A. (2023). Prevention of cardiovascular disease in young adults: Focus on gender differences. A collaborative review from the EAS Young Fellows. Atherosclerosis.

[2] Vos T., Lim S.S., Abbafati C., Abbas K.M., Abbasi M., Abbasifard M., Abbasi-Kangevari M., Abbastabar H., Abd-Allah F., Abdelalim A. (2020). GBD 2019 Diseases and Injuries Collaborators. Global burden of 369 diseases and injuries in 204 countries and territories, 1990–2019: A systematic analysis for the Global Burden of Disease Study 2019. Lancet.

[3] (2020). 2019 Risk Factors Collaborators GBD. Global burden of 87 risk factors in 204 countries and territories, 1990–2019: A systematic analysis for the Global Burden of Disease Study 2019. Lancet.

[4] Lipinski M.J., Escarcega R.O., Lhermusier T., Waksman R. (2014). The effects of novel, bioresorbable scaffolds on coronary vascular pathophysiology. J. Cardiovasc. Transl. Res..

[5] Yanan W., Wu H., Zhongyi Z., Manfred M.F., Kunpeng L., Bo Z., Li Y., Rifang L., Yunbing W. (2022). A thrombin-triggered self-regulating anticoagulant strategy combined with anti-inflammatory capacity for blood-contacting implants. Sci. Adv..

[6] Kanakry C.G. (2021). Probing the entire vascular system. Sci. Transl. Med..

[7] Hansen S., Bui T.A., Xu X., McGrath K. (2025). Preclinical research models for evaluating the biocompatibility of bioresorbable metallic cardiovascular stents: A comparative review. Bioact. Mater..

[8] Aseri M.A., Sundarajoo M., Abidin I.Z., Chee K.H., Selvaraj G., Ghazi A.M., Ahmad W.W. (2026). Percutaneous coronary intervention (PCI) to in-stent restenosis (ISR): Insights from The National Cardiovascular Disease Database—Percutaneous coronary intervention (NCVD-PCI) registry 2021–2022. Int. J. Cardiol..

[9] Lushchik P.E., Botirov M.T., Rafalski I.V., Mamajonov M.M., Niss V.S., Normatova S.A. (2023). Co-Cr alloys for biocompatible medical products. E3S Web of Conferences.

[10] Kapnisis K., Constantinides G., Georgiou H., Cristea D., Gabor C., Munteanu D., Brott B., Anderson P., Lemons J., Anayiotos A. (2014). Multi-scale mechanical investigation of stainless steel and cobalt–chromium stents. J. Mech. Behav. Biomed. Mater..

[11] Liu G., Zhao Y., Li Z., Cao C., Meng J., Yu H., Zhang H. (2023). Investigation of MAF for finishing the inner wall of Super-Slim cardiovascular stents tube. Materials.

[12] Zhang Y., Zhao Y., Fan Q., Yang S., Meng S., Tang Y., Zhang G., Zhang H. (2025). Optimization of Magnetic Finishing Process and Surface Quality Research for Inner Wall of MP35N Cobalt–Chromium Alloy Vascular Stent Tubing Based on Plasma-Fused Al_2_O_3_ Magnetic Abrasives. Micromachines.

[13] Richter B., Radel T., Pfefferkorn F.E. (2022). Sensitivity of surface roughness to laser parameters used for polishing additively manufactured Co-Cr alloy. Surf. Coat. Technol..

[14] (2023). Use of International Standard ISO 10993-1, “Biological Evaluation of Medical Devices--Part 1: Evaluation and Testing Within a Risk Management Process”; Guidance for Industry and Food and Drug Administration Staff; Availability. Fed. Regist./FIND.

[15] (2013). Cardiovascular Implants—Endovascular Instruments—Part 2: Vascular Stents.

[16] Li Z., Zhao Y., Liu G., Cao C., Zhao C., Yu H., Zhang H., Zhao D. (2024). Modeling of material removal by magnetic abrasive finishing of the inner wall of Co-Cr alloy cardiovascular stent tube with diamond magnetic abrasive powder prepared by plasma melting. Int. J. Adv. Manuf. Technol..

[17] Rampal R., Walia A.S., Somani N., Goyal T., Gupta N.K. (2024). Enhancing precision machining: Evaluation of magneto-rheological abrasive finishing (MAF) for brass and SS-304 material using composite abrasives. Int. J. Interact. Des. Manuf..

[18] Qian C., Fan Z., Tian Y., Liu Y., Han J., Wang J. (2021). A review on magnetic abrasive finishing. Int. J. Adv. Manuf. Technol..

[19] Hanada K., Yamaguchi H., Zhou H. (2008). New spherical magnetic abrasives with carried diamond particles for internal finishing of capillary tubes. Diam. Relat. Mater..

[20] Yu S., Liu S., Jiang X., Yang N. (2022). Recent advances on electrochemistry of diamond related materials. Carbon.

[21] Su Z., Zhang S., Liu L.L., Wu J. (2021). Microstructure and performance characterization of Co-based diamond composites fabricated via fused deposition molding and sintering. J. Alloys Compd..

[22] Chen N., Wang R., Nagarajan B., Yan B., Wu Y., He N., Castagne S. (2022). Investigation of metal-coating-assisted IR nanosecond pulsed laser ablation of CVD diamond. J. Mater. Res. Technol..

[23] Song Z., Zhao Y., Li Z., Cao C., Liu G., Liu Q., Zhang X., Dai D., Zheng Z., Zhao C. (2022). Study on the micro removal process of inner surface of cobalt chromium alloy cardiovascular stent tubes. Micromachines.

[24] (1996). Geometrical Product Specifications (GPS)—Surface Texture: Profile Method—Rules and Procedures for the Assessment of Surface Texture.

[25] Song Z., Zhao Y., Liu G., Gao Y., Zhang X., Cao C., Dai D., Deng Y. (2022). Surface roughness prediction and process parameter optimization of Ti-6Al-4 V by magnetic abrasive finishing. Int. J. Adv. Manuf. Technol..

[26] Luo C., Wang X., Duan X., Yang W., Sun S., Li L. (2026). Multi-parameter collaborative optimization and multi-physics coupling mechanisms in double-wall turbine blade cooling structures: A study based on orthogonal experimental design and response surface methodology. Aerosp. Sci. Technol..

[27] Song Z., Zhao Y., Liu G., Cao C., Gao Y., Zhang X., Li Z., Dai D., Pu Y. (2023). Study on finishing inner wall of CoCr alloy cardiovascular stent tube via novel atomized CBN/metal spherical magnetic abrasive powders. J. Manuf. Process..

[28] Marinina O., Nechitailo A., Stroykov G., Tsvetkova A., Reshneva E., Turovskaya L. (2023). Technical and economic assessment of energy efficiency of electrification of hydrocarbon production facilities in underdeveloped areas. Sustainability.

[29] Vapnik V., Levin E., Le Cun Y. (1994). Measuring the VC-dimension of a learning machine. Neural Comput..

